# Adaptive State Observer Design for Dynamic Links in Complex Dynamical Networks

**DOI:** 10.1155/2020/8846438

**Published:** 2020-10-21

**Authors:** Zilin Gao, Jiang Xiong, Jing Zhong, Fuming Liu, Qingshan Liu

**Affiliations:** ^1^Key Laboratory of Intelligent Information Processing and Control of Chongqing Municipal Institutions of Higher Education, Chongqing Three Gorges University, Chongqing 404100, China; ^2^School of Computer Science and Engineering, Chongqing Three Gorges University, Chongqing 404100, China; ^3^School of Mathematics, Southeast University, Nanjing 210009, China

## Abstract

The state observer for dynamic links in complex dynamical networks (CDNs) is investigated by using the adaptive method whether the networks are undirected or directed. In this paper, a complete network model is proposed, which is composed of two coupled subsystems called nodes subsystem and links subsystem, respectively. Especially, for the links subsystem, associated with some assumptions, the state observer with parameter adaptive law is designed. Compared to the existing results about the state observer design of CDNs, the advantage of this method is that a estimation problem of dynamic links is solved in directed networks for the first time. Finally, the results obtained in this paper are demonstrated by performing a numerical example.

## 1. Introduction

In recent past decades, the research on CDNs has become a hot topic in many fields [1–4]. From the perspective of large system, a complete CDN contains many nodes and links (weights of connections between nodes), which implies that a complete CDN is composed of the nodes subsystem and links subsystem, and the two subsystems are usually coupled with each other [5–7]. It is worth noting that the existing researches mainly focus on the nodes subsystem because some behaviors are reflected by nodes such as synchronization [8, 9], stabilization [10, 11], and consensus [12, 13].

From the above results about the synchronization, stabilization, consensus, or other problems of CDNs, it is easy to see all states in CDNs, including the states of nodes and links, are required to be measured accurately. However, this assumption is too hard to be satisfied in practice because of the influence of external environment, measurement costs, and technical constraints [14]. Thus, constructing state observers for the CDNs to estimate the unknown states is very necessary and important. Fortunately, some scholars have discussed the state estimation problems of CDNs and obtained some research results, including cases with the coupling time delays [15, 16], packet loss [17, 18], stochastic noisy disturbance [19], and uncertain coupling strength [20].

However, the above results only consider the estimation problems of the states in nodes subsystem, and assume that the links between nodes are known. It implies that the measurement and state estimation problems of links in the CDNs are ignored. In fact, due to the limitation of measurement methods, the state values of links in CDNs are more difficult to be measured accurately in practical situation, compared to the states of nodes. Hence, only a few papers have studied and discussed the effective measurement problem of the links between individuals (nodes), and the measurement method mainly depends on the physical interaction between individuals [21] or the adaptive weights of links [22]. Similar to the state values of nodes, not all state values of links' weights can be measured and obtained. Therefore, it is necessary to design observers to estimate the unmeasured state values of links. As we know, there is only one paper to have discussed the state estimation problem of dynamic links in CDNs [23]. Unfortunately, the method proposed in [23] is only effective for undirected networks and cannot solve the estimation problem of dynamic links in directed networks.

Inspired by the above discussions, this paper mainly focuses on the state observer design for dynamic links in directed networks. Specifically, a mathematical model for a class of directed CDNs is proposed, which is described by both the nodes subsystem and links subsystem with coupling between the two subsystems, and we have designed a state observer for the links subsystem by using the adaptive method. This means that a state estimation problem of dynamic links in directed networks is solved for the first time, which is also regarded as the biggest contribution of this paper.

The rest of this paper is organized as follows: in Section 2, a complete CDN model is proposed, which is composed of the nodes subsystem and links subsystem with outputs; Section 3 introduces the design process of state observer for the links subsystem; in Section 4, the simulation example is presented and used to demonstrate the effectiveness of this method; finally, the conclusions are given in Section 5.

### 1.1. Notations

The *n*-dimensional Euclidean space is denoted as *R*^*n*^, the set of *n* × *n* real matrices is denoted as *R*^*n*×*n*^, the Euclidean norm of a vector or a matrix is denoted as ‖·‖, and the transpose of matrix *A* and *n*-dimensional identity matrix is denoted as *A*^T^ and *I*_*n*_, respectively.

## 2. Preliminaries and Model Description

If the states of nodes and links in CDNs evolve over time, then the mathematical model of CDNs, including directed and undirected networks, can be described by both the nodes subsystem and links subsystem, where the two subsystems are coupled with each other. In this paper, we only consider the case that each node is *n*-dimensional continuous system in CDNs with *N* nodes, then the nodes subsystem and links subsystem can be described by vector differential equations and matrix differential equation as follows, respectively:

(1) $$\begin{array}{c} {\dot{x}}_{i}=A_{i}x_{i}+B_{i}f_{i}\left(x_{i}\right)+c_{i}\overset{N}{\underset{j=1}{\sum }}p_{ij}\left(t\right)H_{j}\left(x_{j}\right),\;i=1,2,\ldots ,N, \end{array}$$

(2) $$\begin{array}{c} \left\{\begin{array}{c} \dot{P}=\Theta_{1}P+P\Theta_{2}^{\text{T}}+G\left(x\right),\\ Y_{1}=\Upsilon P,Y_{2}=\Upsilon P^{\text{T}}, \end{array}\right. \end{array}$$

where *x*_*i*_=[*x*_*i*1_, *x*_*i*2_,…,*x*_*in*_]^T^ ∈ *R*^*n*^ is the state vector of node *i*; the constant matrices *A*_*i*_ ∈ *R*^*n*×*n*^ and *B*_*i*_ ∈ *R*^*n*×*m*^; the vector functions *f*_*i*_(*x*_*i*_)=[*f*_*i*1_(*x*_*i*_), *f*_*i*2_(*x*_*i*_),…,*f*_*im*_(*x*_*i*_)]^T^ and *H*_*j*_(*x*_*j*_)=[*H*_*j*1_(*x*_*j*_), *H*_*j*2_(*x*_*j*_),…,*H*_*jn*_(*x*_*j*_)]^T^; *c*_*i*_ > 0 is a known constant, which denotes the common connection strength of node *i* in the network; the constant matrices Θ_1_ ∈ *R*^*N*×*N*^ and Θ_2_ ∈ *R*^*N*×*N*^; the coupling matrix *G*(*x*) ∈ *R*^*N*×*N*^, and *x*=[*x*_1_^T^, *x*_2_^T^,…,*x*_*N*_^T^]^T^ ∈ Λ⊆*R*^*Nn*^, where Λ is a bounded and closed set in *R*^*Nn*^; the output matrix *Υ* ∈ *R*^*N*_1_×*N*^ is given; and the links matrix *P*=*P*(*t*)=(*p*_*ij*_(*t*))_*N*×*N*_, where the state variable *p*_*ij*_(*t*) denotes the weight of link from node *j* to node *i*. Especially, *p*_*ji*_=*p*_*ij*_ for undirected networks, and at least, one pair *i*, *j* such that *p*_*ji*_ ≠ *p*_*ij*_ for directed networks. In addition, if *i*=*j*, then *p*_*ij*_ denotes the link strength of node *i* itself.

For the CDNs composed of subsystems (1) and (2), the following instructions are given:

1. The background of dynamic links is given as follows. For example, the biological neural networks consist of neurons (nodes) and synapse (links), and Gamma oscillations in neurons may cause the synaptic facilitation, which is regarded as a dynamic behavior of the links [5, 6, 24]. Similarly, the web winding systems can be regarded to be composed of motors (nodes) and the web (links), and the regulation values of web tensions vary with the speed of the motors, which is also regarded as a dynamic behavior of links [25]. In the above examples, the state values of links need to be measured by some sensors.
2. The existing research results show that the nodes in networks can emerge synchronization or stabilization phenomenon with the help of the links, which mean that the nodes are the main body of synchronization and stabilization [8–11]. In contrast, the links as another part of networks can also emerge some characteristic phenomena in many real networks, such as the structural balance in social networks [5, 6, 26]. It is worth noting that the paper [26] has researched on structural balance by using the Riccati matrix differential equation, and the reason is that this type of equation is more easily to emerge the phenomenon of structural balance. In view of this, we choose linear Riccati matrix differential equation to describe the links subsystem. Clearly, the model of CDNs, composed of both nodes subsystem (1) and links subsystem (2), can help us to understand and explain the dynamic behaviors of networks in a better way.
3. The subsystem (2) is used to describe dynamic change of links' weights in the CDNs, and in general, the CDNs are directed. However, if Θ_1_=Θ_2_ and *G*(*x*)=(*G*(*x*))^T^, then we can obtain *P*=*P*^T^, while the initial value of the state in subsystem (2) satisfies *P*(0)=(*P*(0))^T^. Hence, the subsystems (1) and (2) can be used to describe both undirected and directed networks (the undirected networks can be regarded as a special case of directed networks). To the best of my knowledge, there is only one paper to have solved the state estimation problem of links subsystem [23]. However, this method is only effective for undirected networks, but not for directed networks. This drives us to study estimation problems of dynamic links in directed networks.
4. It is difficult to accurately measure all states of the links between individuals (nodes) in practical applications, which imply that only partial states in (2) can be measured accurately and made available (*N*_1_ < *N*). It is worth noting that the precise measurement of the partial states is bidirectional; that is, if *p*_*ij*_(*t*) is measurable, then *p*_*ji*_(*t*) must also be measurable. That is why the two outputs *Y*_1_ and *Y*_2_ appear in (2).

Now, some useful definitions and operators involved in this paper will be introduced as follows.

**Definition 1 .**

(see [27]). The application vec : *R*^*k*×*l*^⟶*R*^*kl*^ is defined by

(3) $$\begin{array}{c} \text{vec}\left(H\right)={\left[h_{11},\ldots ,h_{1l},h_{21},\ldots ,h_{2l},\ldots ,h_{k1},\ldots ,h_{kl}\right]}^{\text{T}}, \end{array}$$

where the matrix *H*=(*h*_*ij*_)_*k*×*l*_ is called the vectorization operator.

**Definition 2 .**

(see [27]). If there are two matrices *H* ∈ *R*^*k*×*l*^ and *Z* ∈ *R*^*c*×*d*^, then the Kronecker product of *H* and *Z* is denoted as *H* ⊗ *Z* ∈ *R*^*kc*×*ld*^ and defined as follows:

(4) $$\begin{array}{c} H⊗Z=\left(\begin{array}{cccc} h_{11}Z & h_{12}Z & \cdots & h_{1l}Z\\ h_{21}Z & h_{22}Z & \cdots & h_{2l}Z\\ \cdot & \cdot & \cdot & \cdot \\ \cdot & \cdot & \cdot & \cdot \\ h_{k1}Z & h_{k2}Z & \cdots & h_{kl}Z \end{array}\right). \end{array}$$

By using Definitions 1 and 2, the following basic properties about Kronecker product and operator vec(·) can be obtained and shown as follows [27]:

1. (*H* ⊗ *S*)(*X* ⊗ *W*)=(*HX*) ⊗ (*SW*)
2. (*H* ⊗ *W*)^T^=*H*^T^ ⊗ *W*^T^
3. (*S* ⊗ *X*)^−1^=*S*^−1^ ⊗ *X*^−1^
4. vec(*HSW*)=(*H* ⊗ *W*^T^)vec(*S*)
5. vec(*HS*+*SW*)=(*H* ⊗ *I*+*I* ⊗ *W*^T^)vec(*S*)

*S* and *X* are the matrices with compatible dimensions, and *I* represents the identity matrix with compatible dimensions. Especially, it is assumed that both *S* and *X* are invertible in property (3).

According to Definitions 1 and 2 and their corresponding properties, the Riccati differential equation (2) can be rewritten as

(5) $$\begin{array}{c} \left\{\begin{array}{c} \text{vec}\left(\dot{P}\right)=A\text{vec}\left(P\right)+\text{vec}\left(G\left(x\right)\right),\\ \text{vec}\left(Y_{1}\right)=C_{1}\text{vec}\left(P\right),\text{vec}\left(Y_{2}\right)=C_{1}\text{vec}\left(P^{\text{T}}\right), \end{array}\right. \end{array}$$

where *A*=Θ_1_ ⊗ *I*_*N*_+*I*_*N*_ ⊗ Θ_2_ and *C*_1_=Υ ⊗ *I*_*N*_.

**Assumption 1 .**

For the links subsystem (2), the double matrices (Θ_1_, Υ) and (Θ_2_, Υ) are completely stable.

If Assumption 1 is true, then we can obtain matrices *K*_1_ ∈ *R*^*N*×*N*_1_^ and *K*_2_ ∈ *R*^*N*×*N*_1_^, which can make Θ_1_+*K*_1_Υ and Θ_2_+*K*_2_Υ to be Hurwitz stable, respectively. Thus, as long as any matrices *Q*_1_ > 0 and *Q*_2_ > 0 are given, there must be positive definite matrices *M*_1_ ∈ *R*^*N*×*N*^ and *M*_2_ ∈ *R*^*N*×*N*^ that satisfy the following two Lyapunov equations, respectively:

(6) $$\begin{array}{c} {\left(\Theta_{1}+K_{1}\Upsilon \right)}^{\text{T}}M_{1}+M_{1}\left(\Theta_{1}+K_{1}\Upsilon \right)=-Q_{1}, \end{array}$$

(7) $$\begin{array}{c} {\left(\Theta_{2}+K_{2}\Upsilon \right)}^{\text{T}}M_{2}+M_{2}\left(\Theta_{2}+K_{2}\Upsilon \right)=-Q_{2}. \end{array}$$

**Lemma 1 .**

If Assumption 1 is true, then the following Lyapunov equations

(8) $$\begin{array}{c} {\left(\Theta_{1}⊗I_{N}+{\tilde{K}}_{1}C_{1}\right)}^{\text{T}}\tilde{M}+\tilde{M}\left(\Theta_{1}⊗I_{N}+{\tilde{K}}_{1}C_{1}\right)=-{\tilde{Q}}_{1},\\ {\left(I_{N}⊗\Theta_{2}+{\tilde{K}}_{2}C_{2}\right)}^{\text{T}}\tilde{M}+\tilde{M}\left(I_{N}⊗\Theta_{2}+{\tilde{K}}_{2}C_{2}\right)=-{\tilde{Q}}_{2}, \end{array}$$

hold, where $\tilde{M}=M_{1}⊗M_{2}$, ${\tilde{Q}}_{1}=Q_{1}⊗M_{2}$, ${\tilde{Q}}_{2}=M_{1}⊗Q_{2}$, ${\tilde{K}}_{1}=K_{1}⊗I_{N}$, ${\tilde{K}}_{2}=I_{N}⊗K_{2}$, and *C*_2_=*I*_*N*_ ⊗ Υ. Clearly, $\tilde{M}>0$, ${\tilde{Q}}_{1}>0$, and ${\tilde{Q}}_{2}>0$.

Proof. If Assumption 1 holds, then the following equations can be obtain from (6) and (7):

(9) $$\begin{array}{c} \left[{\left(\Theta_{1}+K_{1}\Upsilon \right)}^{\text{T}}M_{1}\right]⊗I_{N}+\left[M_{1}\left(\Theta_{1}+K_{1}\Upsilon \right)\right]⊗I_{N}=-Q_{1}⊗I_{N}, \end{array}$$

(10) $$\begin{array}{c} I_{N}⊗\left[{\left(\Theta_{2}+K_{2}\Upsilon \right)}^{\text{T}}M_{2}\right]+I_{N}⊗\left[M_{2}\left(\Theta_{2}+K_{2}\Upsilon \right)\right]=-I_{N}⊗Q_{2}. \end{array}$$

Using the properties of Kronecker product, (9) and (10) can be rewritten as

(11) $$\begin{array}{c} {\left[\Theta_{1}⊗I_{N}+\left(K_{1}⊗I_{N}\right)\left(\Upsilon ⊗I_{N}\right)\right]}^{\text{T}}\left(M_{1}⊗I_{N}\right)\\ +\left(M_{1}⊗\right.\left.I_{N}\right)\left[\Theta_{1}⊗I_{N}+\left(K_{1}⊗I_{N}\right)\left(\Upsilon ⊗I_{N}\right)\right]=-Q_{1}⊗I_{N},\\ {\left[I_{N}⊗\Theta_{2}+\left(I_{N}⊗K_{2}\right)\left(I_{N}⊗\Upsilon \right)\right]}^{\text{T}}\left(I_{N}⊗M_{2}\right)\\ +\left(I_{N}⊗\right.\left.M_{2}\right)\left[I_{N}⊗\Theta_{2}+\left(I_{N}⊗K_{2}\right)\left(I_{N}⊗\Upsilon \right)\right]=-I_{N}⊗Q_{2}. \end{array}$$

Thus, we can get

(12) $$\begin{array}{c} {\left[\Theta_{1}⊗I_{N}+{\tilde{K}}_{1}C_{1}\right]}^{\text{T}}\left(M_{1}⊗I_{N}\right)+\left(M_{1}⊗I_{N}\right)\left[\Theta_{1}⊗I_{N}+{\tilde{K}}_{1}C_{1}\right]=-Q_{1}⊗I_{N}, \end{array}$$

(13) $$\begin{array}{c} {\left[I_{N}⊗\Theta_{2}+{\tilde{K}}_{2}C_{2}\right]}^{\text{T}}\left(I_{N}⊗M_{2}\right)+\left(I_{N}⊗M_{2}\right)\left[I_{N}⊗\Theta_{2}+{\tilde{K}}_{2}C_{2}\right]=-I_{N}⊗Q_{2}. \end{array}$$

If we multiply both sides of the equalities (12) and (13) by (*I*_*N*_ ⊗ *M*_2_) and (*M*_1_ ⊗ *I*_*N*_) from right, respectively, then we get that

(14) $$\begin{array}{c} {\left[\Theta_{1}⊗I_{N}+{\tilde{K}}_{1}C_{1}\right]}^{\text{T}}\left(M_{1}⊗I_{N}\right)\left(I_{N}⊗M_{2}\right)\\ +\left(M_{1}⊗I_{N}\right)\left[\Theta_{1}⊗I_{N}+{\tilde{K}}_{1}C_{1}\right]\left(I_{N}⊗M_{2}\right)=-\left(Q_{1}⊗I_{N}\right)\left(I_{N}⊗M_{2}\right), \end{array}$$

(15) $$\begin{array}{c} {\left[I_{N}⊗\Theta_{2}+{\tilde{K}}_{2}C_{2}\right]}^{\text{T}}\left(I_{N}⊗M_{2}\right)\left(M_{1}⊗I_{N}\right)\\ +\left(I_{N}⊗M_{2}\right)\left[I_{N}⊗\Theta_{2}+{\tilde{K}}_{2}C_{2}\right]\left(M_{1}⊗I_{N}\right)=-\left(I_{N}⊗Q_{2}\right)\left(M_{1}⊗I_{N}\right). \end{array}$$

It is noticed that (*M*_1_ ⊗ *I*_*N*_)(*I*_*N*_ ⊗ *M*_2_)=*M*_1_ ⊗ *M*_2_=(*I*_*N*_*M*_1_) ⊗ (*M*_2_*I*_*N*_)=(*I*_*N*_ ⊗ *M*_2_)(*M*_1_ ⊗ *I*_*N*_). Therefore, the equalities (14) and (15) can be rewritten as follows:

(16) $$\begin{array}{c} {\left[\Theta_{1}⊗I_{N}+{\tilde{K}}_{1}C_{1}\right]}^{\text{T}}\left(M_{1}⊗M_{2}\right)+\left(M_{1}⊗M_{2}\right)\left[\Theta_{1}⊗I_{N}+{\tilde{K}}_{1}C_{1}\right]=-Q_{1}⊗M_{2},\\ {\left[I_{N}⊗\Theta_{2}+{\tilde{K}}_{2}C_{2}\right]}^{\text{T}}\left(M_{1}⊗M_{2}\right)+\left(M_{1}⊗M_{2}\right)\left[I_{N}⊗\Theta_{2}+{\tilde{K}}_{2}C_{2}\right]=-M_{1}⊗Q_{2}. \end{array}$$

Thus, Lemma 1 is completely proved.

**Assumption 2 .**

For subsystem (2), in which the coupling matrix *G*(*x*) satisfies that *G*(*x*)=*M*_1_^−1^Υ^T^Ψ(*x*)*M*_2_^−1^, where Ψ(*x*)=(*ψ*_*ij*_)_*N*_1_×*N*_ and *ψ*_*ij*_=*x*_*i*_^T^*x*_*j*_.

If Assumption 2 holds, then we can get that $\left\|\Psi \left(x\right)\right\|=\sqrt{\sum_{i=1}^{N_{1}}\sum_{j=1}^{N}{\left(x_{i}^{\text{T}}x_{j}\right)}^{2}}\leq \sqrt{\sum_{i=1}^{N_{1}}\sum_{j=1}^{N}{\left(\left\|x_{i}\right\|\cdot \left\|x_{j}\right\|\right)}^{2}}$ ≤ $\sqrt{\sum_{i=1}^{N}{\left\|x_{i}\right\|}^{2}\sum_{j=1}^{N}{\left\|x_{j}\right\|}^{2}}={\left\|x\right\|}^{2}$. Meanwhile, we note that Λ is a bounded and closed set in *R*^*Nn*^, and *x* ∈ Λ. Thus, there exists a positive constant *L* to satisfy the inequality ‖*x*‖^2^ ≤ *L*.

General speaking, *L* is unknown. However, we can use the adaptive method to estimate it. In this paper, we use $\hat{L}=\hat{L}\left(t\right)$ to denote the estimated value of *L*. Hence, the estimation error is denoted as $\tilde{L}=\hat{L}-L$.

## 3. Main Results

**Definition 3 .**

Designing a matrix differential system $\dot{\hat{P}}=F\left(\hat{P},Y_{1},Y_{2},\hat{L}\right)$, if the state $\hat{P}$ satisfies $\lim_{t⟶+\infty }\left(P-\hat{P}\right)=0$, then the matrix differential system $\dot{\hat{P}}=F\left(\hat{P},Y_{1},Y_{2},\hat{L}\right)$ can be regarded as a state observer of the links subsystem (2).

If Assumptions 1 and 2 hold, the state observer of the links subsystem (2) can be designed and presented as follows:

(17) $$\begin{array}{c} \dot{\hat{P}}=\left(\Theta_{1}+K_{1}\Upsilon \right)\hat{P}+\hat{P}{\left(\Theta_{2}+K_{2}\Upsilon \right)}^{\text{T}}+\Gamma \left(\hat{P},Y_{1},Y_{2},\hat{L}\right)-K_{1}Y_{1}-Y_{2}^{\text{T}}K_{2}^{\text{T}}, \end{array}$$

with the following adaptive law

(18) $$\begin{array}{c} \dot{\hat{L}}=\frac{1}{\rho }\left\|\text{vec}\left(Y_{1}\right)-C_{1}\text{vec}\left(\hat{P}\right)\right\|, \end{array}$$

where $\hat{P}$ denotes the estimated value of the state *P* in (2); the robust term $\Gamma \left(\hat{P},Y_{1},Y_{2},\hat{L}\right)=\left\{\begin{array}{c} \Omega ,\Upsilon \hat{P}\neq Y_{1}\\ 0,\Upsilon \hat{P}=Y_{1} \end{array}\right.$, where $\Omega =\hat{L}\left(\left(M_{1}^{-1}\Upsilon^{\text{T}}\left(Y_{1}-\Upsilon \hat{P}\right)M_{2}^{-1}\right)|/|\left(\left\|Y_{1}-\Upsilon \hat{P}\right\|\right)\right)$, *ρ* is a given positive constant, and the matrices *K*_1_, *K*_2_, *M*_1_, and *M*_2_ can be obtained by solving the Lyapunov equations (6) and (7), respectively.

According to (3) and (4), we can deduce from (17) that

(19) $$\begin{array}{c} \text{vec}\left(\dot{\hat{P}}\right)=\left(A+{\tilde{K}}_{1}C_{1}+{\tilde{K}}_{2}C_{2}\right)\text{vec}\left(\hat{P}\right)+\text{vec}\left(\Gamma \left(\hat{P},Y_{1},Y_{2},\hat{L}\right)\right)-{\tilde{K}}_{1}\text{vec}\left(Y_{1}\right)-{\tilde{K}}_{2}\text{vec}\left(Y_{2}^{\text{T}}\right). \end{array}$$

Clearly, $\left\|Y_{1}-\Upsilon \hat{P}\right\|=\left\|Y_{1}^{\text{T}}-{\hat{P}}^{\text{T}}\Upsilon^{\text{T}}\right\|=\left\|\text{vec}\left(Y_{1}-\Upsilon \hat{P}\right)\right\|=\left\|\text{vec}\left(Y_{1}^{\text{T}}-{\hat{P}}^{\text{T}}\Upsilon^{\text{T}}\right)\right\|$; thus, we get $\text{vec}\left(\Omega \right)=\hat{L}\left(\left({\tilde{M}}^{-1}C_{1}^{\text{T}}\left[\text{vec}\left(Y_{1}\right)-C_{1}\text{vec}\left(\hat{P}\right)\right]\right)|/|\left(\left\|\text{vec}\left(Y_{1}\right)-C_{1}\text{vec}\left(\hat{P}\right)\right\|\right)\right)$.

In this paper, the estimation error is denoted by $E=P-\hat{P}$. By using (3), (4), and properties about Kronecker product and vec(·) operator, we can get the following error system:

(20) $$\begin{array}{c} \text{vec}\left(\dot{E}\right)=\left(A+{\tilde{K}}_{1}C_{1}+{\tilde{K}}_{2}C_{2}\right)\text{vec}\left(E\right)+{\tilde{M}}^{-1}C_{1}^{\text{T}}\text{vec}\left(\Psi \left(x\right)\right)-\text{vec}\left(\Gamma \left(\hat{P},Y_{1},Y_{2},\hat{L}\right)\right)\;. \end{array}$$

**Theorem 1 .**

If Assumptions 1 and 2 are true, then the matrix differential system (17) with the parameter adaptive law (18) is the state observer of the links subsystem (2).

Proof. Consider the following Lyapunov function:

(21) $$\begin{array}{c} V=\frac{1}{2}\text{vec}{\left(E\right)}^{\text{T}}\tilde{M}\text{vec}\left(E\right)+\frac{1}{2}\rho {\tilde{L}}^{2}. \end{array}$$

Calculating the orbit derivative of *V* along (20) gives that

(22) $$\begin{array}{c} \dot{V}\;=\text{ vec}{\left(E\right)}^{\text{T}}\tilde{M}\text{vec}\left(\dot{E}\right)+\rho \tilde{L}\dot{\hat{L}}\\ =\text{vec}{\left(E\right)}^{\text{T}}\tilde{M}\left\{\left(A+{\tilde{K}}_{1}C_{1}+{\tilde{K}}_{2}C_{2}\right)\text{vec}\left(E\right)\right.+\left.{\tilde{M}}^{-1}C_{1}^{\text{T}}\text{vec}\left(\Psi \left(x\right)\right)-\text{vec}\left(\Gamma \left(\hat{P},Y_{1},Y_{2},\hat{L}\right)\right)\right\}+\rho \tilde{L}\dot{\hat{L}}\\ =\text{vec}{\left(E\right)}^{\text{T}}\tilde{M}\left(\Theta_{1}⊗I_{N}+{\tilde{K}}_{1}C_{1}\right)\text{vec}\left(E\right)+\text{vec}{\left(E\right)}^{\text{T}}\tilde{M}\left(I_{N}⊗\Theta_{2}+{\tilde{K}}_{2}C_{2}\right)\text{vec}\left(E\right)\\ +\text{vec}{\left(E\right)}^{\text{T}}C_{1}^{\text{T}}\text{vec}\left(\Psi \left(x\right)\right)+\rho \tilde{L}\dot{\hat{L}}-\left\{\begin{array}{c} \begin{array}{c} \hat{L}\frac{\text{vec}{\left(E\right)}^{\text{T}}C_{1}^{\text{T}}\left[C_{1}\text{vec}\left(E\right)\right]}{\left\|C_{1}\text{vec}\left(E\right)\right\|},C_{1}\text{vec}\left(\hat{P}\right)\neq \text{vec}\left(Y_{1}\right)\\ 0,C_{1}\text{vec}\left(\hat{P}\right)=\text{vec}\left(Y_{1}\right) \end{array} \end{array}\right.\\ \leq \frac{1}{2}\text{vec}{\left(E\right)}^{\text{T}}\left[{\left(\Theta_{1}⊗I_{N}+{\tilde{K}}_{1}C_{1}\right)}^{\text{T}}\tilde{M}\right.+\left.\tilde{M}|\left(\Theta_{1}⊗I_{N}+{\tilde{K}}_{1}C_{1}\right)\right]\text{vec}\left(E\right)\\ +\frac{1}{2}\text{vec}{\left(E\right)}^{\text{T}}\left[{\left(I_{N}⊗\Theta_{2}+{\tilde{K}}_{2}C_{2}\right)}^{\text{T}}\tilde{M}\right.+\left.\tilde{M}|\left(I_{N}⊗\Theta_{2}+{\tilde{K}}_{2}C_{2}\right)\right]\text{vec}\left(E\right)\\ +\left\|\text{vec}{\left(E\right)}^{\text{T}}C_{1}^{\text{T}}\right\|\left\|\text{vec}\left(\Psi \left(x\right)\right)\right\|+\rho \tilde{L}\dot{\hat{L}}-\left\{\begin{array}{c} \hat{L}\left\|\text{vec}{\left(E\right)}^{\text{T}}C_{1}^{\text{T}}\right\|,C_{1}\text{vec}\left(\hat{P}\right)\neq \text{vec}\left(Y_{1}\right)\\ 0,C_{1}\text{vec}\left(\hat{P}\right)=\text{vec}\left(Y_{1}\right) \end{array}\right.\\ \leq -\frac{1}{2}\text{vec}{\left(E\right)}^{\text{T}}\left({\tilde{Q}}_{1}+{\tilde{Q}}_{2}\right)\text{vec}\left(E\right)+L\left\|\text{vec}{\left(E\right)}^{\text{T}}C_{1}^{\text{T}}\right\|+\rho \tilde{L}\dot{\hat{L}}\\ -\left\{\begin{array}{c} \hat{L}\left\|\text{vec}{\left(E\right)}^{\text{T}}C_{1}^{\text{T}}\right\|,C_{1}\text{vec}\left(\hat{P}\right)\neq \text{vec}\left(Y_{1}\right)\\ 0,C_{1}\text{vec}\left(\hat{P}\right)=\text{vec}\left(Y_{1}\right) \end{array}\right.\\ =-\frac{1}{2}\text{vec}{\left(E\right)}^{\text{T}}\left({\tilde{Q}}_{1}+{\tilde{Q}}_{2}\right)\text{vec}\left(E\right)+\rho \tilde{L}\dot{\hat{L}}+\hat{L}\left\|\text{vec}{\left(E\right)}^{\text{T}}C_{1}^{\text{T}}\right\|-\tilde{L}\left\|\text{vec}\left(Y_{1}\right)-C_{1}\text{vec}\left(\hat{P}\right)\right\|\\ -\left\{\begin{array}{c} \hat{L}\left\|\text{vec}{\left(E\right)}^{\text{T}}C_{1}^{\text{T}}\right\|,C_{1}\text{vec}\left(\hat{P}\right)\neq \text{vec}\left(Y_{1}\right)\\ 0,C_{1}\text{vec}\left(\hat{P}\right)=\text{vec}\left(Y_{1}\right) \end{array}\right.\\ =-\frac{1}{2}\text{vec}{\left(E\right)}^{\text{T}}\left({\tilde{Q}}_{1}+{\tilde{Q}}_{2}\right)\text{vec}\left(E\right)+\tilde{L}\left(\rho \dot{\hat{L}}-\left\|\text{vec}\left(Y_{1}\right)-C_{1}\text{vec}\left(\hat{P}\right)\right\|\right)\\ =-\frac{1}{2}\text{vec}{\left(E\right)}^{\text{T}}\left({\tilde{Q}}_{1}+{\tilde{Q}}_{2}\right)\text{vec}\left(E\right). \end{array}$$

From inequality (22), we can obtain that the estimation error matrix *E* is bounded and $E\overset{t⟶+\infty }{⟶}0$. Thus, Theorem 1 is completely proved.

## 4. Simulation Example

In this paper, we consider a continuous analog Hopfield network with 10 neurons (*N*=10) [23, 28], which is composed of nodes subsystem and links subsystem, where the nodes subsystem is described as follows:

(23) $$\begin{array}{c} {\dot{x}}_{i}=A_{i}x_{i}+B_{i}f_{i}\left(x_{i}\right)+c_{i}\overset{10}{\underset{j=1}{\sum }}p_{ij}H_{j}\left(x_{j}\right),\;i=1,2,\ldots ,10, \end{array}$$

where *A*_*i*_=*B*_*i*_=−*i*, *f*_*i*_(*x*_*i*_)=−5cos*t*, *c*_*i*_=*i*, and *H*_*j*_(*x*_*j*_)=(1 − *e*^−*x*_*j*_^)/(1+*e*^−*x*_*j*_^).

Meanwhile, we assume that the changes in the links' weights *p*_*ij*_(*t*) satisfy the Riccati differential equation (2). If we choose *N*_1_=5 and *ρ*=100 and randomly select matrices Θ_1_ ∈ *R*^10×10^,  Θ_2_ ∈ *R*^10×10^, and *Υ* ∈ *R*^5×10^ satisfying Assumption 1, then the matrices *K*_1_, *M*_1_ and *K*_2_, *M*_2_ can be obtained by solving the Lyapunov equations (6) and (7), respectively. Thus, we can get the coupling matrix *G*(*x*)=*M*_1_^−1^Υ^T^Ψ(*x*)*M*_2_^−1^ in (2) satisfying Assumption 2.

**Figure 1:**
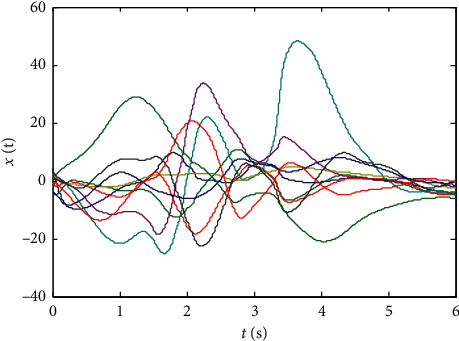
State trajectories of subsystem (1).

**Figure 2:**
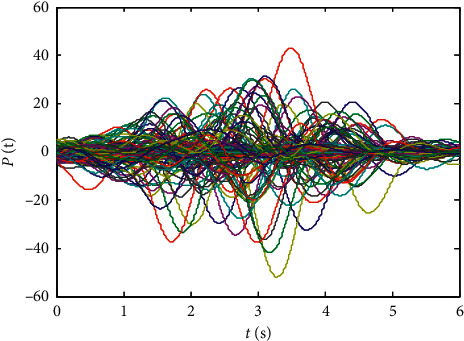
State trajectories of subsystem (2).

**Figure 3:**
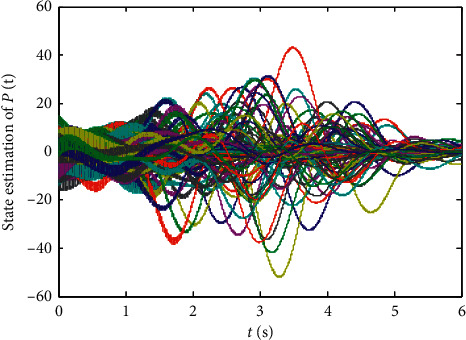
State trajectories of state observer (17).

**Figure 4:**
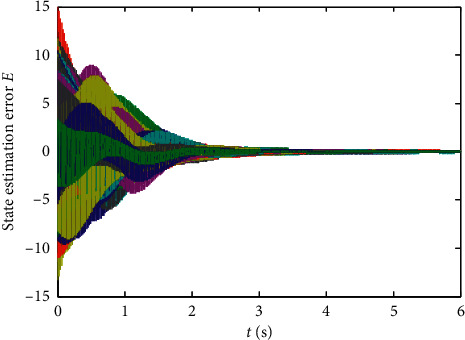
State trajectories of estimation error system (20).

**Figure 5:**
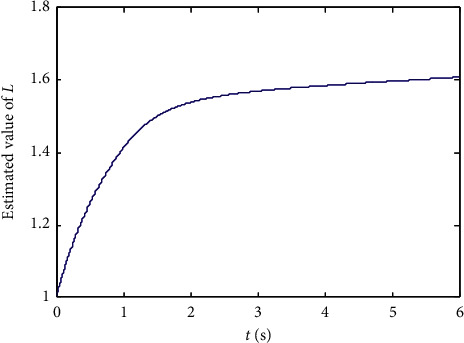
State trajectories of parameter adaptive system (18).

Finally, randomly select the initial values of states $x_{i}\left(0\right),\hat{L}\left(0\right)$, and *p*_*ij*_(0), *i*, *j*=1,2,…, 10 in the range (−5,5), and the numerical results are shown in Figures 1–5:

- From Figures 2–4, we can see that the estimation error converges asymptotically to zero. According to Definition 3, we know that the Riccati dynamical equation (17) with the adaptive law (18) is a state observer of the subsystem (2), and the state observer is effective.
- Compared to the results in [23], our advantage is that the result about the state observer of the subsystem (2) is true whatever the network is directed or undirected. Meanwhile, it is worth noting that, due to the effect of the parameter adaptive law (18), the state observer (17) does not contain the states of the nodes. This shows that the state observer is less affected by the dynamic changes in the nodes and thus improves the robustness of the state observer.

## 5. Conclusions

In this paper, a complete model of CDNs is proposed, which is composed of two coupled subsystems, called nodes subsystem and links subsystem, respectively. Contrary to the existing results on the state estimation problem of nodes subsystem, we mainly focus on the state estimation of the links subsystem with outputs and have designed a state observer with the parameter adaptive law to estimate the state of the links subsystem in this paper. In particular, this method solves the estimation problem of dynamic links in directed networks for the first time. Meanwhile, it implies that we can use the state estimation information of the links to directly design a controller for the links subsystem; thus, some control problems may be solved effectively. Therefore, the design method of state observer for dynamic links proposed in this paper can enrich the achievements about the state estimation of CDNs.

## Data Availability

In this paper, we submitted data mainly related to theoretical proof and numerical simulation, in which the part of numerical simulation is realized by Matlab software; if necessary, we can provide simulation source program and relevant data at any time.
